# Supplementary material for: Functional and structural asymmetry suggest a unifying principle for catalysis in membrane-bound pyrophosphatases
Source: EMBO Rep. 2024 Jan 5;25(2):853–75. doi: 10.1038/s44319-023-00037-x (PMC10897367; doi:10.1038/s44319-023-00037-x)
Supplement: Supplementary file 1 — Table EV1 [file 44319_2023_37_MOESM1_ESM.pdf]

Table EV 1: X-ray data collection and refinement statistics of 3.8 Å *Pa*PPase:Mg_5IDP structure.

**Data collection**	
Space group	$P2_1$
Cell dimensions	
a, *b*, *c* (Å)	107.2, 88.0, 116.8
α, β, γ (°)	90.0, 106.9, 90.0
Source	DLS: i04/i24<br>ESRF: ID23-1/MASSIF1
Wavelength* (Å)	0.966/0.979/0.971/0.968
Resolution (Å)	19.97-3.84 (3.98-3.84)
Overall (Å)	3.8
along h axis	5.3
along k axis	4.1
along l axis	3.8
Measured reflections	65003 (2063)
Unique reflections	13069 (653)
Completeness (%)	87.6
$CC_{1/2}$	0.979
Mean I/σ(I)	3.9
Multiplicity	5.0
B-factors ($Å^2$)	112.11
R_{merge}	0.335 (1.524)
R_{meas}	0.375 (1.749)
R_{pim}	0.163 (0.827)
**Refinement**	
R_{work}(%)/R_{free}(%)	28.9/31.1
No. of atoms	9734
Protein	9694
Ligands	33
Water	7
No. of chains (ASU)	2
B-factors ($Å^2$)	106.96
Protein	106.98
Ligands/Ions	94.85F
R. M. S. Deviations	
Bond lengths (Å)	0.002
Bond angle (°)	0.047
Ramachandran statistics (%)	
Favoured	96.61
Allowed	3.16
Outliers	0.23

Statistics for the highest-resolution shell are shown in parentheses
* Data from several beamlines
