# Supplementary material for: Functional and structural asymmetry suggest a unifying principle for catalysis in membrane-bound pyrophosphatases
Source: EMBO Rep. 2024 Jan 5;25(2):853–75. doi: 10.1038/s44319-023-00037-x (PMC10897367; doi:10.1038/s44319-023-00037-x)
Supplement: Supplementary file 2 — Table EV2 [file 44319_2023_37_MOESM2_ESM.pdf]

**Table EV2: X-ray data collection and refinement statistics of time-resolved *Tm*PPase structures at 0, 0-60, 300, 600 and 3600 seconds post-activation with $Na^{+}$.**

| Data collection | t = 0 s | t = 0-60 s | t = 300 s | t = 600 s | t = 3600 s |
|---|---|---|---|---|---|
| Dataset`* | Single best | Grouped | Combined | Combined | Combined |
| Space group | $P2_1$ | $P2_1$ | $P2_1$ | $P2_1$ | $P2_1$ |
| Cell dimensions | | | | | |
| *a*, *b*, *c* (Å) | 84.0, 110.2, 108.0 | 84.0, 110.1, 107.4 | 83.9, 110.6, 106.1 | 83.3, 111.6, 106.2 | 83.1, 111.1, 105.4 |
| α, β, γ (°) | 90.0, 107.9, 90.0 | 90.0, 108.0, 90.0 | 90.0, 108.2, 90.0 | 90.0, 109.1, 90.0 | 90.0, 108.9, 90.0 |
| Source | DESY: P14-I | DESY: P14-I | DESY: P14-I | DESY: P14-I | DESY: P14-I |
| Wavelength (Å) | 0.976 | 0.976 | 0.976 | 0.976 | 0.976 |
| Resolution (Å) | 75.34-2.65 (3.08-2.65) | 102.13-2.57 (2.89-2.57) | 100.81-3.97 (4.09-3.97) | 100.4-3.84 (4.17-3.84) | 99.74-4.53 (5.22-4.53) |
| Overall (Å) | 2.65 | 2.54 | 3.97 | 3.84 | 4.53 |
| along h axis | 2.65 | 2.54 | 3.77 | 3.86 | 4.53 |
| along k axis | 3.32 | 2.95 | 3.79 | 3.84 | 5.40 |
| along l axis | 3.79 | 3.38 | 4.51 | 4.71 | 6.00 |
| Measured reflections | 194305 (9787) | 1793856 (27736) | 90019 (2490) | 85342 (2342) | 43682 (2211) |
| Unique reflections | 27956 (1398) | 37297 (1868) | 14163 (708) | 13078 (654) | 6063 (303) |
| Completeness (%) | 91.6 | 93.8 | 95.7 | 90.7 | 87.8 |
| $CC_{1/2}$ | 0.999 | 0.996 | 0.975 | 0.965 | 0.995 |
| Mean I/σ(I) | 12.3 | 12.2 | 6.1 | 4.4 | 10.1 |
| Multiplicity | 7.0 | 48.1 | 6.4 | 6.5 | 7.2 |
| B-factors ($Å^2$) | 108.65 | 70.28 | 88.03 | 210.38 | 286.11 |
| $R_{merge}$ | 0.064 (0.993) | 0.265 (3.733) | 0.187 (0.589) | 0.165 (0.779) | 0.066 (1.307) |
| $R_{meas}$ | 0.069 (1.073) | 0.268 (3.867) | 0.203 (0.697) | 0.179 (0.918) | 0.072 (1.409) |
| $R_{pim}$ | 0.026 (0.042) | 0.037 (0.995) | 0.079 (0.368) | 0.067 (0.479) | 0.027 (0.519) |
| **Refinement** | **t = 0 s** | **t = 0-60 s** | **t = 300 s** | **t = 600 s** | **t = 3600 s** |
| Search Model | PDB: 4AV3 | PDB: 4AV3 | t=0-60 s | Chain A: 5LZQ (A); Chain B: t=0-60 s (B) | Chain A: 5LZQ (A); Chain B: t=0-60 s (B) |
| Active site | Empty | Empty | A: $Mg_4PP_i$, B: - | A: $Mg_4PP_i$, B: - | A: $Mg_5PP_i$, B: $Mg_4P_{i\,2}$ |
| $R_{work}$(%)/$R_{free}$(%) | 23.8/27.4 | 21.95/23.61 | 23.53/26.37 | 33.42/36.34 | 32.59/36.06 |
| No. of atoms | 10330 | 10715 | 10522 | 10077 | 10275 |
| Protein | 10311 | 10502 | 10508 | 10063 | 10246 |
| Ligands/Lipids | 0 | 212 | 13 | 13 | 29 |
| Water | 13 | 1 | 1 | 1 | 0 |
| No. of chains (per ASU) | 2 | 2 | 2 | 2 | 2 |
| B-factors ($Å^2$) | 114.96 | 79.30 | 132.51 | 215.75 | 406.84 |
| Protein | 114.99. | 79.29 | 132.43 | 215.84 | 406.81 |
| Ligands/Ions | 103.14 | 79.86 | 195.19 | 192.68 | 416.95 |
| R. M. S. Deviations | | | | | |
| Bond lengths (Å) | 0.001 | 0.003 | 0.003 | 0.005 | 0.003 |
| Bond angle (°) | 0.35 | 0.57 | 0.60 | 0.81 | 0.62 |
| Ramachandran statistics (%) | | | | | |
| Favoured | 97.86 | 96.90 | 97.04 | 97.88 | 98.72 |
| Allowed | 2.14 | 3.10 | 2.96 | 2.12 | 1.28 |
| Outliers | 0.00 | 0.00 | 0.37 | 0.00 | 0.00 |

Statistics for the highest-resolution shell are shown in parentheses
* Single best: dataset from best diffracting crystal, grouped: datasets from different time-points, combined: datasets from the same time-points
