# Supplementary material for: Functional and structural asymmetry suggest a unifying principle for catalysis in membrane-bound pyrophosphatases
Source: EMBO Rep. 2024 Jan 5;25(2):853–75. doi: 10.1038/s44319-023-00037-x (PMC10897367; doi:10.1038/s44319-023-00037-x)
Supplement: Supplementary file 3 — Appendix [file 44319_2023_37_MOESM3_ESM.pdf]

Appendix

Table of Figures **Page**

Appendix Figure S1:	“Hot-solve” purification and analytical SEC of M-PPases.	**2**
Appendix Figure S2:	Electron density maps of *Pa*PPase:Mg_5IDP key regions.	**3**
Appendix Figure S3:	$2mF_o$-dF_c: electron density map of the K^+/$K^{12.46}$ cationic centre of *Pa*PPase:Mg_5IDP.	**3**
Appendix Figure S4:	Orientation of helix 5, helix 13 and helix 10 (opposing subunit) in different IDP-bound M-PPase structures.	**4**
Appendix Figure S5:	Pre-studies of time-resolved crystallographic experiments with *Tm*PPase.	**4**
Appendix Figure S6:	Kinetic model for the catalysis by M-PPase homodimeric enzyme.	**5**

List of Tables

Appendix Table S1:	M-PPase numbering scheme.	**6**
Appendix Table S2:	Helix by helix comparison of *Pa*PPase:Mg_5IDP structure to other M-PPase structures.	**7**
Appendix Table S3:	HELANAL-Plus curvature analysis of helix 5 of *Pa*PPase:Mg_5IDP.	**7**
Appendix Table S4:	Comparison of the hydrogen bonding pattern around $S^{5.43}$ and D6.43 in IDP-bound structures.	**8**
Appendix Table S5:	Rotamer options for $K^{12.46}$ in *Pa*PPase:Mg_5IDP of the backbone-independent Richardson library.	**8**
Appendix Table S6:	Rotamer options for $K^{16.50}$ *Pa*PPase:Mg_5IDP of the backbone-independent Richardson library.	**9**
Appendix Table S7:	Catalytic turnover of wild-type *Tm*PPase used for time-resolved structural studies in various conditions upon NaCl activation	**9**

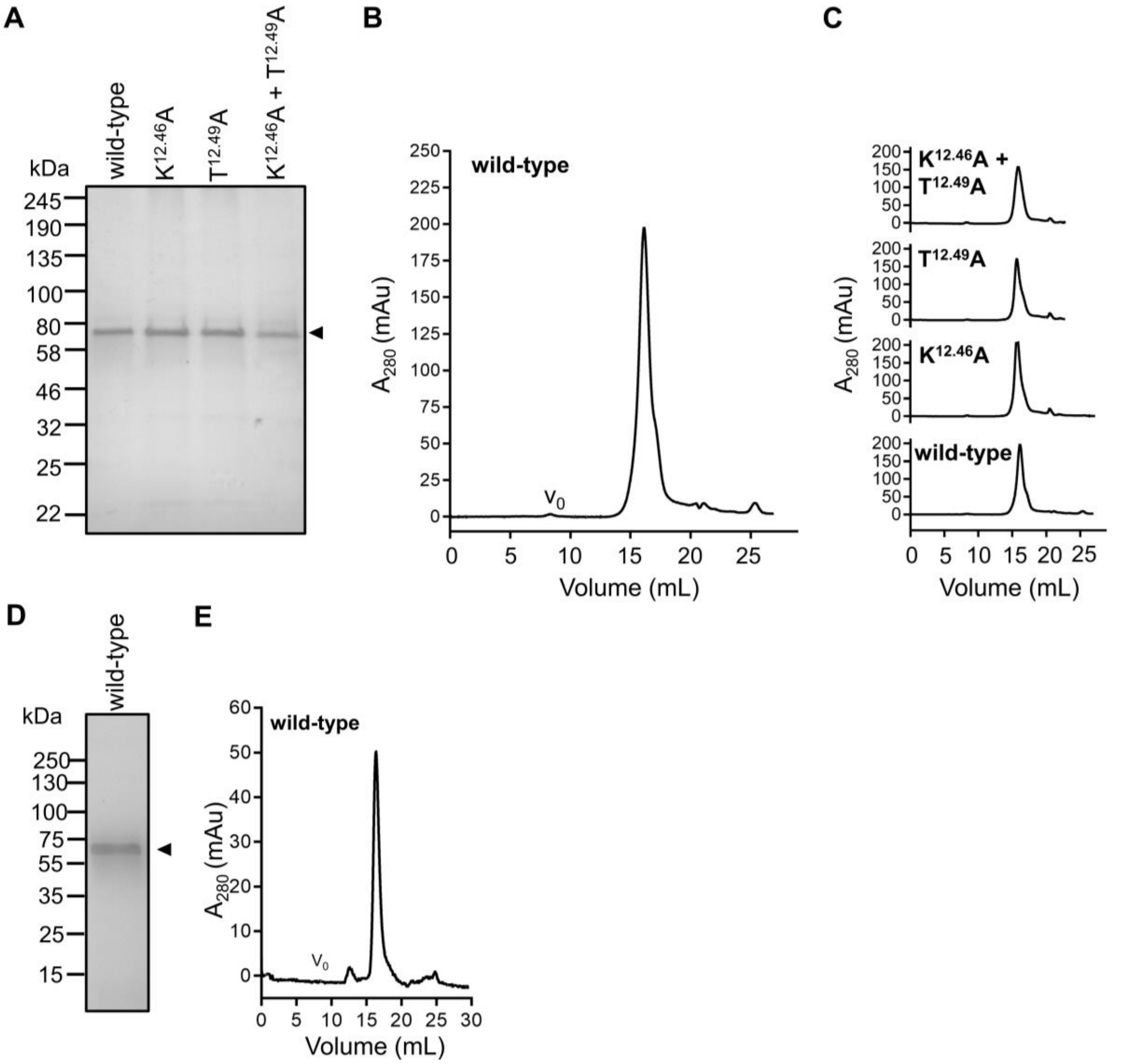

Appendix Figure S1: “Hot-solve” purification and analytical SEC of M-PPases. (A) SDS-PAGE (Coomassie stain) analysis of purified wild-type and variant *Pa*PPase. **(B)** Analytical SEC of wild-type *Pa*PPase on Superose 6 Increase 10/300 column. The void volume is indicated by v_o. **(C)** SEC elution volume comparison of wild-type and variant *Pa*PPase on Superose 6 Increase 10/300 column. **(D)** SDS-PAGE (Coomassie stain) analysis of purified wild-type *Tm*PPase. **(E)** Analytical SEC of wild-type *Tm*PPase on Superose 6 Increase 10/300 column. The void volume is indicated by v_o.

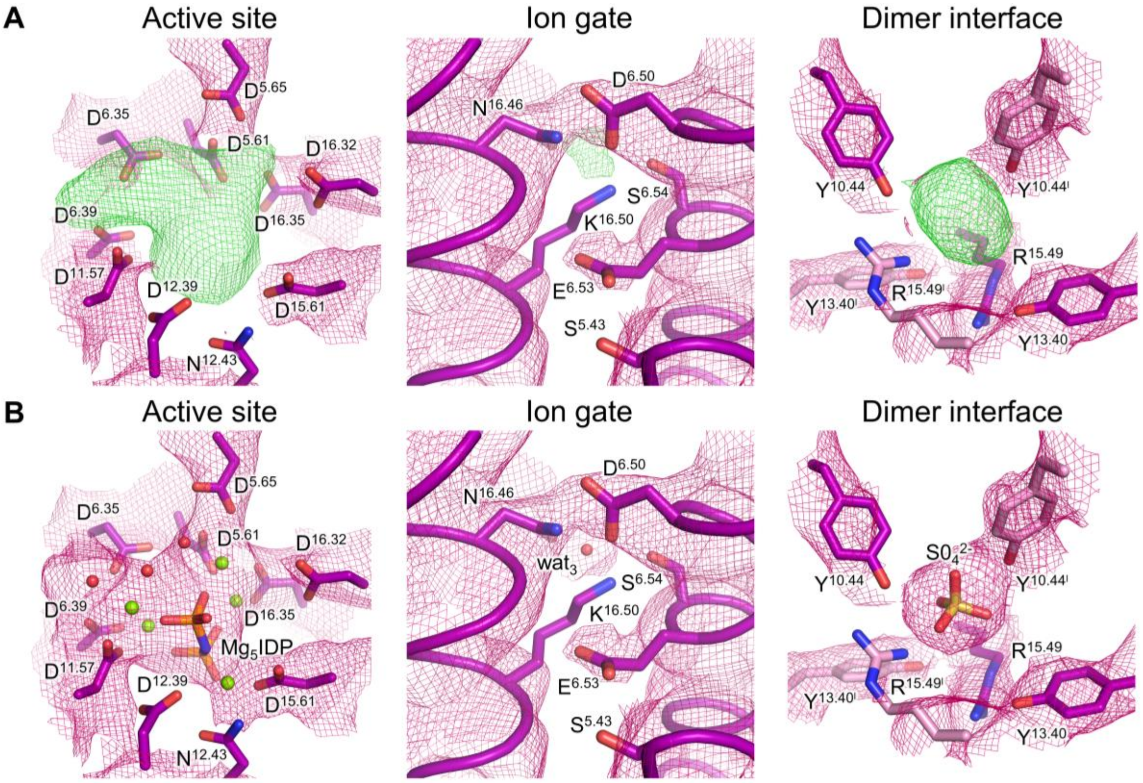

Appendix Figure S2: Electron density maps of *Pa*PPase:Mg_5IDP key regions. (A) $2mF_o$-dF_c map of active site, ion gate and dimer interface residues and mF_o-dF_c omit map with positive density shown in green and negative density shown in red for ligand or heteroatom binding regions. **(B)** $2mF_o$-dF_c map of active site, ion gate and dimer interface residues with ligands and heteroatoms added to the model. Mg^{2+} are shown as green spheres and structural water molecules are shown as red spheres. Residues of subunit A are coloured in purple and residues of subunit B a coloured in pink (additionally marked with apostrophes). $2mF_o$-dF_c density is shown at 1 σ and mF_o-dF_c is shown at 3 σ.

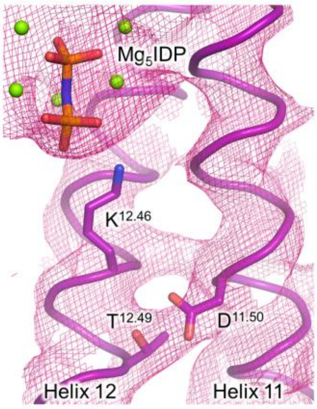

Appendix Figure S3: $2mF_o$-dF_c: electron density map of the K^+/$K^{12.46}$ cationic centre of *Pa*PPase:Mg_5IDP. $2mF_o$-dF_c: electron density of key residue $K^{12.46}$ and nearby residues $T^{12.49}$ and $D^{11.50}$ are shown at 3 σ.

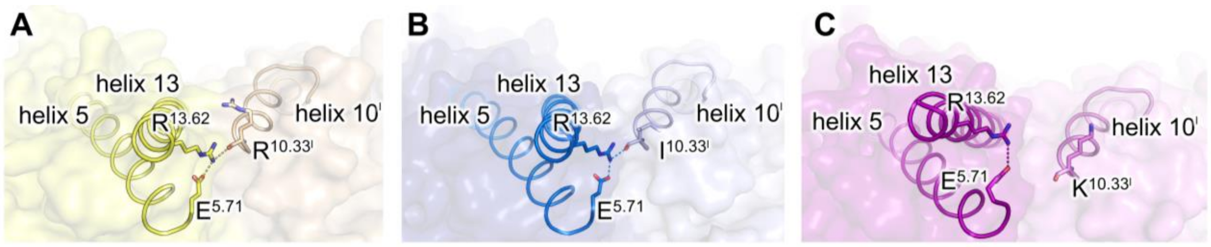

Appendix Figure S4: Orientation of helix 5, helix 13 and helix 10 (opposing subunit) in different IDP-bound M-PPase structures. (A) Orientation shown in *Vr*PPase:Mg_5IDP (PDB: 4A01). **(B)** Orientation shown in *Tm*PPase:Mg_5IDP (PDB: 5LZQ). **(C)** Orientation shown in *Pa*PPase:Mg_5IDP (this study). Salt bridge network interactions of $E^{5.71}$-$R^{13.62}$-R/$K^{10.33'}$ are represented by dashed lines in both panels. Subunit A and B are coloured differently, and residues of subunit B are marked by an apostrophe.

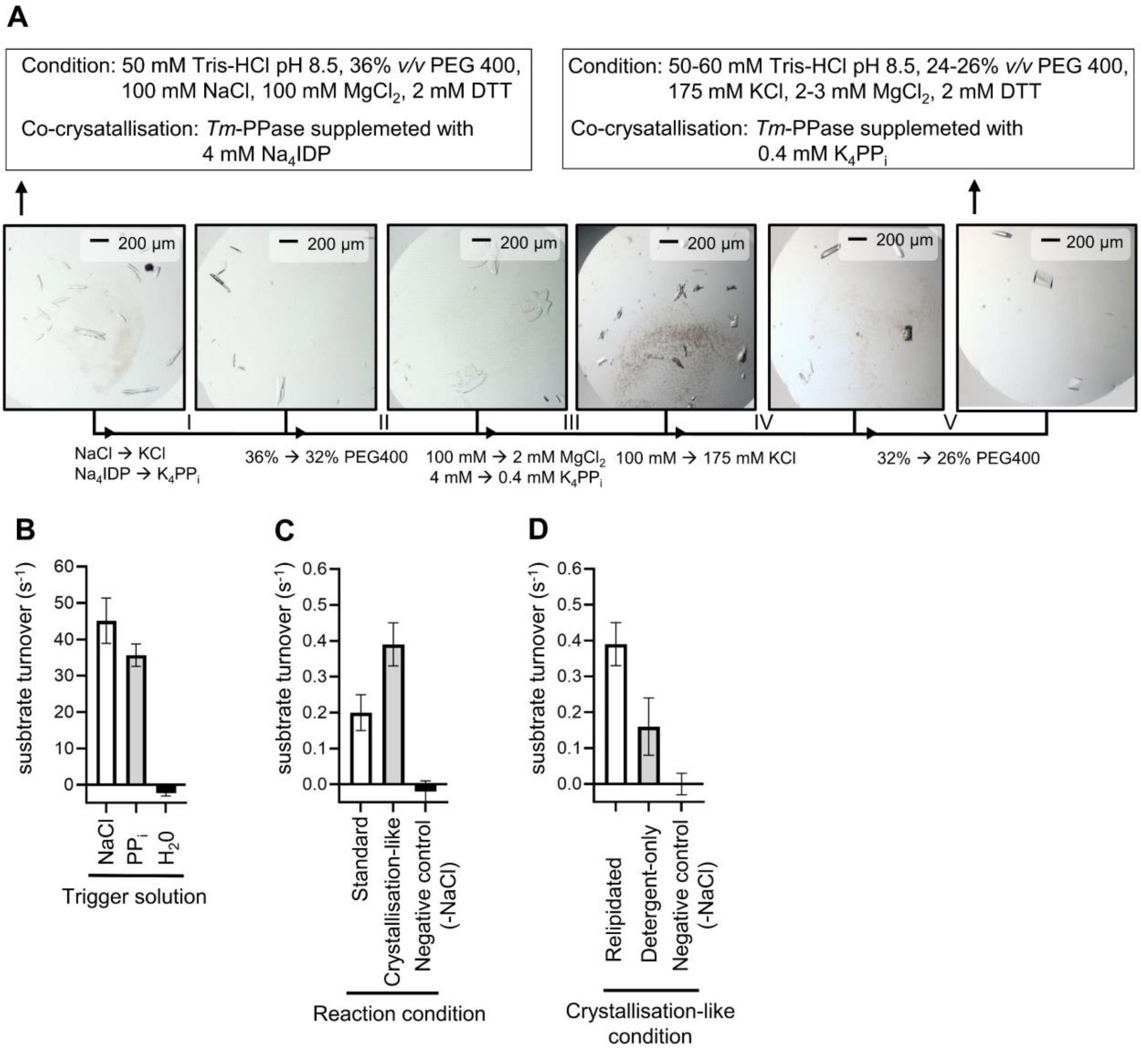

Appendix Figure S5: Pre-studies of time-resolved crystallographic experiments with *Tm*PPase. (A) Crystal optimisation rounds I-V. Arrows indicate the optimisation steps and changes are annotated. . The start and end condition are displayed in black boxes. **(B-D)** Quantitative P_i-release activity assays of *Tm*PPase in a range of different reaction conditions. **(B)** Substrate turnover by *Tm*PPase at 71 °C upon reaction initiation with different trigger solutions. **(C)** Substrate turnover by *Tm*PPase at 20 °C in different reaction conditions. **(D)** Substrate turnover by *Tm*PPase at 20 °C in crystallisation-like reaction conditions after different sample treatments. Negative controls lack NaCl in the final reaction condition (-NaCl). Error is shown as SEM of three technical repeats.

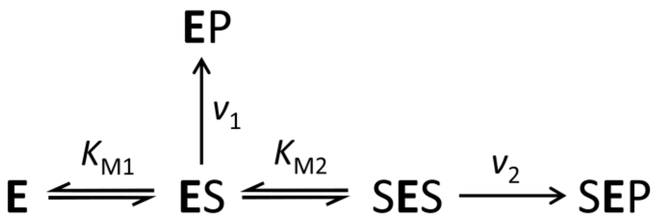

Appendix Figure S6. Kinetic model for the catalysis by M-PPase homodimeric enzyme.

Appendix Table S1: M-PPase numbering scheme.

B&W‡	*Tm*PPase	*Vr*PPase	*Pa*PPase
1.50	F17	F25	Y20
2.50	K55	K94	R58
3.50	S87	S153	S96
4.50	G130	G194	G138
5.43	S184	S235	S176
5.50	R191	R242	R183
5.61	D202	D253	D194
5.65	D206	D257	D198
5.71	E212	E263	E204
6.35	D228	D279	D220
6.39	D232	D283	D224
6.43	D236	D287	D228
6.47	L240	M291	M232
6.50	D243	D294	D235
6.53	E246	G297	E238
6.54	S247	S298	S239
6.57	G250	R301	V242
7.50	G294	G335	A271
8.50	L321	L361	I294
9.50	G369	G411	G333
10.33	I399	R441	K363
10.44	L410	L452	Y374
10.50	S416	S458	S380
11.50	D458	D500	D439
11.57	D465	D507	D446
12.39	D488	D530	D469
12.43	N492	N534	N473
12.46	A495	A537	K476
12.49	G498	G540	T479
12.50	K499	K541	K480
13.40	Y556	Y587	Y544
13.50	V566	V597	V554
13.62	R578	R609	R566
14.50	M611	M642	F599
15.49	L648	I679	R636
15.50	A649	A680	A637
15.61	D660	D691	D48
16.31	D688	D723	D672
16.35	D692	D727	D676
16.38	K695	K730	K679
16.39	D696	D731	D680
16.46	D703	N738	N687
16.50	K707	K742	K691

‡ Ballesteros & Weinstein nomenclature (Ballesteros & Weinstein, 1995)

Appendix Table S2: Helix by helix comparison of *Pa*PPase:Mg_5IDP structure to other M-PPase structures.

R.m.s.d. (Å) *	*Pa*PPase:Mg_5IDP *versus* †							5LZQ *vs.* 4A01†
	5LZQ	4A01	4AV3	4AV6	5GPJ	6AFS	Average	
Active site status	Active: Substrate-analogue bound	Active: Substrate-analogue bound	Resting: Inhibitory Ca^{2+} bound	Resting: Product bound	Resting: Product bound	Resting: Product bound		
Overall	1.24	1.50	1.67	1.236	1.50	1.49	1.44	0.86
Helix 1	2.16	2.70	2.31	2.23	2.21	2.72	2.39	4.70
Helix 2	1.33	2.29	1.43	1.66	2.41	2.42	1.92	0.86
Helix 3	1.37	2.06	1.93	1.33	1.57	2.06	1.72	4.78
Helix 4	1.18	1.26	1.79	1.58	1.57	1.30	1.45	1.98
Helix 5	1.79	1.61	1.79	1.46	1.09	1.37	1.52	1.41
Helix 6	1.59	1.18	1.32	1.44	0.89	1.10	1.25	1.35
Helix 7	1.84	2.38	2.28	2.07	2.34	2.39	2.22	1.68
Helix 8	1.87	2.09	2.97	2.06	1.81	2.06	2.14	0.96
Helix 9	1.25	1.48	1.85	1.46	1.63	1.48	1.52	1.05
Helix 10	1.20	1.79	1.43	1.09	1.78	1.77	1.51	2.39
Helix 11	1.10	1.43	1.52	1.23	1.34	1.37	1.33	1.92
Helix 12	0.91	0.94	2.77	1.13	2.05	0.94	1.45	0.68
Helix 13 ‡	3.01	3.26	1.01	1.03	1.71	3.23	2.21	1.27
Helix 14 ‡	2.94	2.73	1.78	1.87	1.85	2.73	2.32	0.70
Helix 15	0.83	0.97	1.30	0.86	1.12	0.95	1.01	0.73
Helix 16	1.44	1.42	1.33	1.26	1.35	1.40	1.37	1.04
Inner ring helices	1.28	1.26	1.67	1.23	1.31	1.19	1.28	-
Outer ring helices	1.82	2.20	1.88	1.64	1.89	2.21	1.82	-

*Structural alignment based on Cα atoms of subunit A.

† PDB identifier column header. 5LZQ: *Tm*PPase:Mg_5IDP, 4AV6: *Tm*PPase:Mg_4P_{i2}, 4AV3: *Tm*PPase:CaMg, 4A01: *Vr*PPase:Mg_5IDP, 5GPJ: *Vr*PPase:Mg_2P_i, 6AFS: *Vr*PPase: Mg_5P_{i2}

‡ *Pa*PPase:Mg_5IDP helices cut to length to helices from comparison structures for alignment

Appendix Table S3: HELANAL-Plus curvature analysis of helix 5 of *Pa*PPase:Mg_5IDP.

**HELANAL-Plus Parameters***	**Helix 5 *Pa*PPase:Mg_5IDP**	**Helix 5 *Vr*PPase:Mg_5IDP**
Helix length (residues)	37	37
Average number of residues per turn	3.66	3.69
Average unit hight of helix (Å)	1.5	1.53
Average virtual torsion angle (°)	49.8	49.8
Average bending angle (°)	11.9	11
Maximum bending angle (°)	24.5	23.2
Radius of sphere curvature *(*Å*)*	114	76
R.m.s.d. of sphere fit (Å) (r.m.s.d.S)	0.247	0.266
R.m.s.d. of linear fit (Å) (r.m.s.d.L)	0.154	0.238
Geometry†	linear	kinked

* Definition of all parameters can be found in (Kumar & Bansal, 2012).

† Classification: Linear if, r.m.s.d.S > r.m.s.d.L and maximum bending angle (MBA) < 20° and if the 20° <MBA< 30°, r.m.s.d.S and r.m.s.d.L are both <0.14 and 0.16 Å, respectively, and r.m.s.d.S > r.m.s.d.L. Kinked, if the value of the MBA is >30° or the MBA is between 20° and 30° and r.m.s.d. to sphere (r.m.s.d.S) and 3D line (r.m.s.d.L) fit is more than 0.14 and 0.16 Å, respectively.

Appendix Table S4: Comparison of the hydrogen bonding pattern around S[5.43] and D[6.43] in IDP-bound structures.

Residue from	Helical geometry*		
	*Pa*PPase:Mg_5IDP	*Vr*PPase:Mg_5IDP	*Tm*PPase:Mg_5IDP
5.37	(α)	3_{10}	3_{10}
5.38	3_{10}	α	3_{10}
5.39	3_{10}	α	(α)
5.40	(α)	α	(α)
5.41	α	α	α
5.42	3_{10}	α	α
5.43	α	α	α
5.43	3_{10}	3_{10}	α
5.44	α	α	α
5.45	(α)	α	α
5.46	α	α	α
6.40	α	(α)	3_{10}
6.41	3_{10}	α	3_{10}
6.42	α	α	α
6.43	α	(α)	(α)
6.44	(α)	π	π
6.45	π	π	π
6.46	π	α	α
6.47	α	3_{10}	π

* Hydrogen bonding from amino nitrogen to upstream carbonyl oxygen of main chain
Entries in parentheses are based on rise per residue as no hydrogen bond (distance > 4 Å) is formed

Appendix Table S5: Rotamer options for K[12.46] in *Pa*PPase:Mg_5IDP of the backbone-independent Richardson library. Sorting based on their vdW radii overlap to surrounding atoms from lowest to highest. Modelled rotamer at the top of the table.

Chi_1	Chi_2	Chi_3	Chi_4	Sum vdW radii overlap [Å]	Clashes*	Hydrogen bonds
62	180	68	180	0.62	1	3
62	180	-68	180	2.00	2	4
63	-178	178	-179	2.55	3	2
63	-170	-177	72	2.84	3	1
62	180	180	-65	3.03	3	1
-70	-179	-66	-64	4.59	4	2
-70	-170	-66	-175	5.96	5	3
179	59	163	60	7.35	7	0
-177	180	68	65	7.46	7	1
-59	-69	-176	-70	7.53	8	3
-58	-61	-177	-179	8.31	10	2
-69	164	62	-179	8.44	7	2
180	179	78	179	8.50	9	4
-67	-176	174	76	9.04	8	0
-177	178	179	180	9.35	9	1
-177	68	180	-65	9.62	8	1
-62	-68	180	65	9.74	10	1
-177	180	171	63	10.01	10	2
-90	68	180	180	10.23	10	2
179	172	178	-72	10.36	8	1
-177	62	173	171	11.62	11	2
-67	-179	-179	-63	12.45	11	1
-59	-58	-75	-174	12.62	12	1
-175	-174	-69	179	12.94	10	2

The chi angles translate to m for minus (-60°), t for trans (±180°) and p for plus (+60°) per chi angle in Coot
* vdW radii overlap of ≥ 0.6 Å is classified as a clash

Appendix Table S6: Rotamer options for $K^{16.50}$ *Pa*PPase:Mg_5IDP of the backbone-independent Richardson library. Sorting based on their vdW radii overlap to surrounding atoms from lowest to highest. Modelled rotamer at the top of the table.

Chi_1	Chi_2	Chi_3	Chi_4	Sum vdW radii overlap [Å]	Clashes*	Hydrogen bonds
-70	-170	-66	-175	0	0	4
-69	164	62	-179	0	0	1
-69	-179	70	67	0	0	0
-177	180	-68	-65	0.83	1	0
-175	-174	-69	179	2.17	3	0
-70	-179	-66	-64	3.08	3	2
-59	-58	-75	-174	3.97	3	1
-67	176	179	177	6.41	5	2
-177	68	180	-65	8.98	9	1
62	180	68	180	9.03	7	4
-67	-179	-179	-63	9.1	8	0
-67	-176	174	76	10.38	9	2
-177	178	179	180	10.63	11	1
-177	180	68	65	11.13	7	1
179	62	173	171	11.68	12	0
-62	-68	180	65	12.41	10	1
179	59	163	60	12.62	12	0
-177	172	178	-72	12.77	11	1
-177	180	171	63	12.85	12	0
-58	-61	-177	-179	13.73	10	2
-59	-69	-176	-70	15.19	11	0
180	179	78	179	15.78	11	0
63	-178	178	-179	17.19	14	1
62	180	180	-65	19.7	15	1

The chi angles translate to m for minus (-60°), t for trans (±180°) and p for plus (+60°) per chi angle in Coot

* vdW radii overlap of ≥ 0.6 Å is classified as a clash

Appendix Table S7: Catalytic turnover of wild-type *Tm*PPase used for time-resolved structural studies in various conditions upon NaCl activation.

Fixed-time P_i release assays	Wild-type *Tm*PPase			
Reaction condition	Standard	Standard	Crystallisation-like	Crystallisation-like
Sample treatment	Relipidated	Relipidated	Relipidated	Detergent-only
Temperature (°C)	71	20	20	20
k_{cat} $(s^{-1})^*$	45.13±3.59	0.20±0.03	0.39±0.03	0.16±0.05

* Error is shown as SEM of three technical repeats.
