# Supplementary material for: Functional and structural asymmetry suggest a unifying principle for catalysis in membrane-bound pyrophosphatases
Source: EMBO Rep. 2024 Jan 5;25(2):853–75. doi: 10.1038/s44319-023-00037-x (PMC10897367; doi:10.1038/s44319-023-00037-x)
Supplement: Supplementary file 9 — Expanded View Figures [file 44319_2023_37_MOESM9_ESM.pdf]

# Expanded View Figures

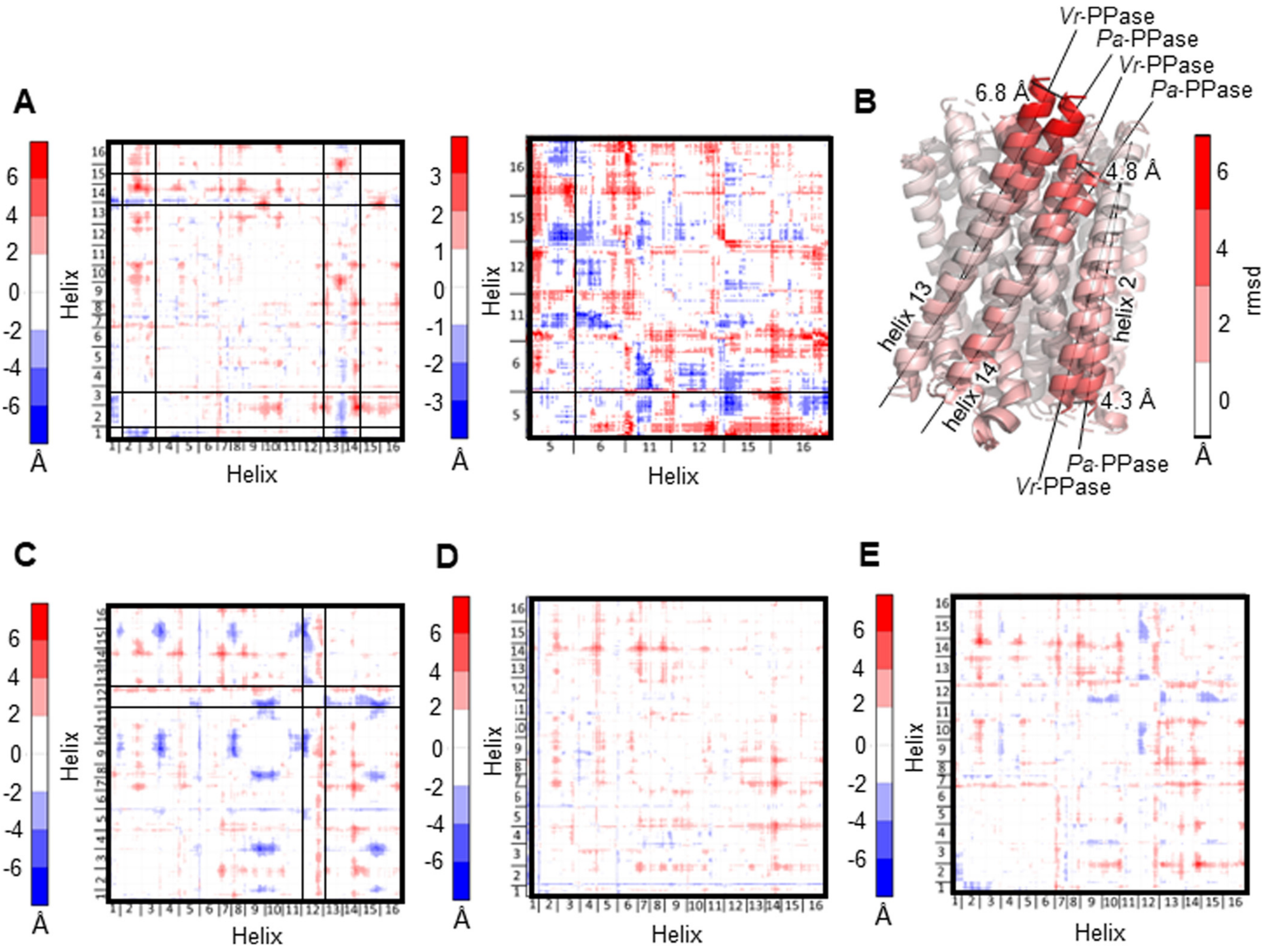


**Figure EV1. Comparison of inter-Cα distances between *Pa*PPase:$Mg_5$IDP and other M-PPase structures.**

The difference in inter-Cα distances is coloured from red (biggest difference) to blue (smallest difference) in each selection and helices with large clusters of changes are highlighted by black boxes. (**A**) Difference distance matrix (DiDiMa) of *Pa*PPase:$Mg_5$IDP (this study) versus *Vr*PPase:$Mg_5$IDP (PDB: 4A01). Left panel shows the DiDiMa of all atoms (scale: ±6 Å); right panel shows inter-atom differences of inner ring helices only (scale: ±3 Å). (**B**) Structural alignment of subunit A of the *Pa* and *Vr*PPase $Mg_5$IDP complexes, with helices coloured by their r.m.s.d./$C_\alpha$. Dashed lines indicate the distances measured at the end of the helices. (**C–E**) DiDiMa (scale: ±6 Å) of *Pa*PPase:$Mg_5$IDP (this study) versus (**C**) *Tm*PPase:CaMg (PDB: 4AV3); (**D**) *Tm*PPase:$Mg_4P_{i2}$ (PDB: 4AV6) and (**E**) *Vr*PPase:$Mg_2P_i$ (PDB: 5GPJ).

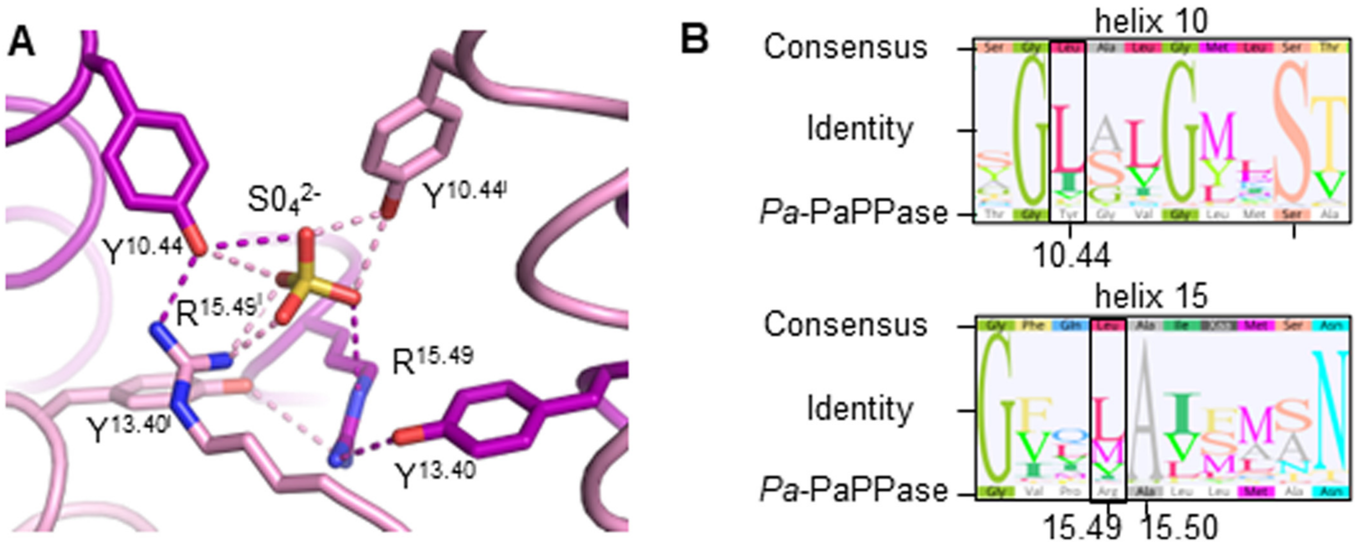


**Figure EV2. $SO_4^{2-}$ binding site at the dimer interface of *Pa*PPase:Mg$_5$IDP.**

(**A**) Structural overview with subunit A in purple and subunit B in pink (additionally marked with apostrophes). Side-chain interactions are shown as dashed lines. (**B**) Sequence analysis of the $SO_4^{2-}$ binding site. The consensus sequence and sequence identity (sequence logo showing the graphical representation of the residue conservation) are based on an alignment of 45 homologous sequences to *Pa*PPase identified in a blastp search of the UniProt database. Residues of interest are highlighted by a black box and labelled following the B&W convention.

 © The Author(s)

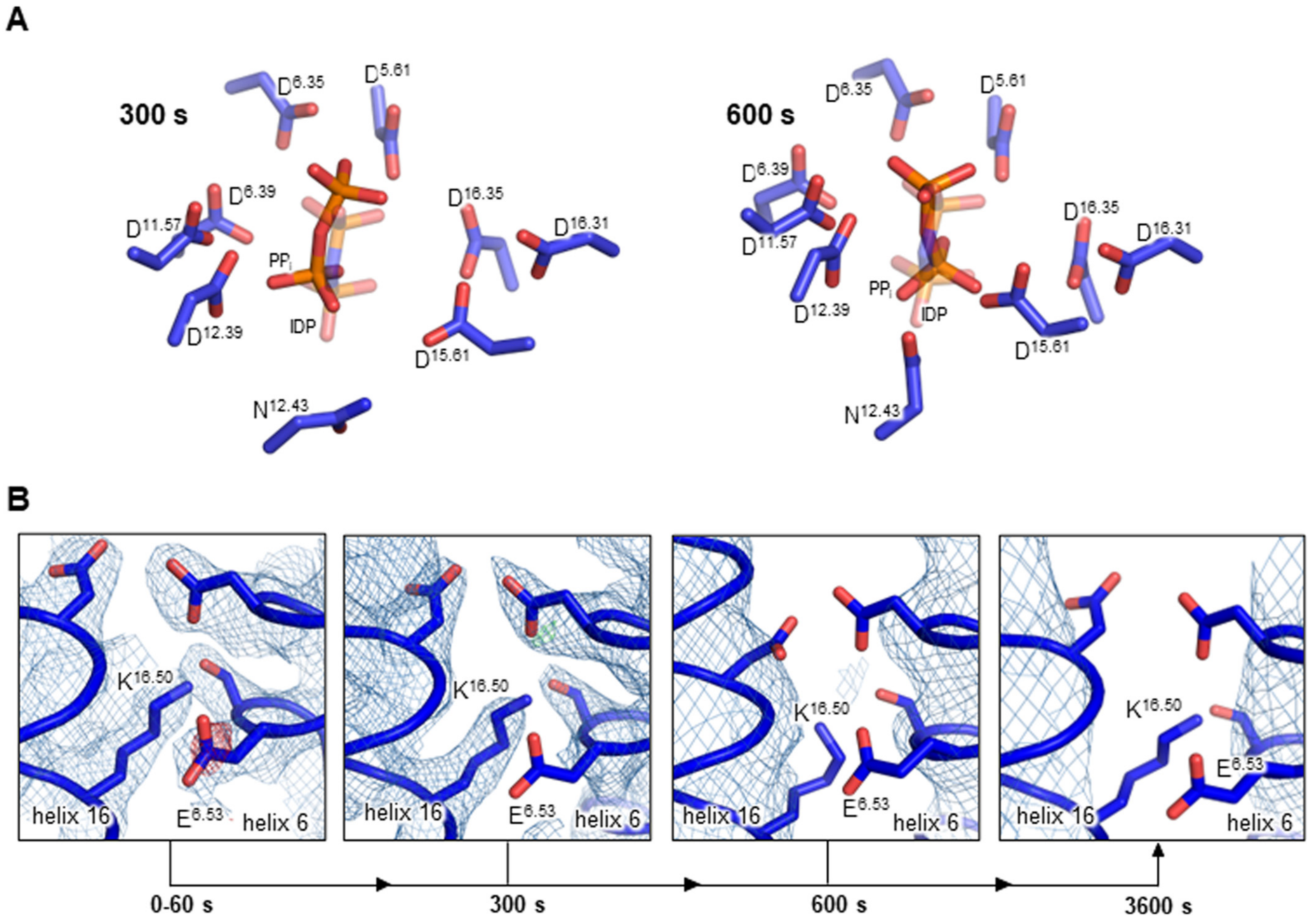


**Figure EV3. Key regions of time-resolved *Tm*PPase structures at different time points.**

(**A**) Comparison of ligand binding at the asymmetrically occupied active site in time-resolved structures after 300 s and 600 s reaction initiation (blue) and the symmetrically occupied active site in the static *Tm*PPase:$Mg_5$IDP structure (transparent, PDB: 5LZQ). The time-resolved structures have physiologically relevant $PP_i$ bound whereas the *Tm*PPase:$Mg_5$IDP structure has the non-hydrolysable substrate analogue IDP-bound. (**B**) Ion gate of time-resolved *Tm*PPase structures of grouped datasets of different time points (0–60 s) and combined datasets of the same time point (300, 600 and 3600 s). Structures show subunit A with $2mF_o$-$dF_c$ density (blue) and $mF_o$-$dF_c$ density (red/green) at 1 σ and 3 σ, respectively. At low $Na^+$ concentrations (0–60 s), the semi-conserved glutamate appears disordered as indicated by negative $mF_o$-$dF_c$ density.
